# Comparative analysis of bone turnover markers in bone marrow and peripheral blood: implications for osteoporosis

**DOI:** 10.1186/s13018-024-04634-x

**Published:** 2024-03-01

**Authors:** Chuan Jiang, Sibo Zhu, Wanda Zhan, Linbing Lou, Aoying Li, Jun Cai

**Affiliations:** 1 College of Medicine, Yangzhou University, Yangzhou, 225001 China; 2 Department of Orthopedics, Dalian Medical University, Dalian, 116000 China; 3 Department of Orthopedics, Clinical Medical College, Yangzhou University, Yangzhou, 225001 China

**Keywords:** BTMs, Bone marrow, Peripheral blood, Osteoporosis, Bone mineral density

## Abstract

**Introduction:**

This study examines bone turnover marker (BTM) variations between bone marrow and peripheral blood in osteoporotic and non-osteoporotic patients. BTMs offer insights into bone remodeling, crucial for understanding osteoporosis.

**Methods:**

A total of 133 patients were categorized into osteoporotic and non-osteoporotic cohorts. BTMs—C-telopeptide cross-linked type 1 collagen (β-CTX), serum osteocalcin (OC), Procollagen type I N-propeptide (P1NP), 25(OH)D—were measured in bone marrow and peripheral blood. Lumbar spine bone mineral density (BMD) was assessed.

**Results:**

Osteoporotic patients exhibited elevated β-CTX and OC levels in peripheral blood, indicating heightened bone resorption and turnover. β-CTX levels in osteoporotic bone marrow were significantly higher. Negative correlations were found between peripheral blood β-CTX and OC levels and lumbar spine BMD, suggesting their potential as osteoporosis severity indicators. No such correlations were observed with bone marrow markers. When analyzing postmenopausal women separately, we obtained consistent results.

**Conclusions:**

Elevated β-CTX and OC levels in osteoporotic peripheral blood highlight their diagnostic significance. Negative β-CTX and OC-BMD correlations underscore their potential for assessing osteoporosis severity. Discrepancies between peripheral blood and bone marrow markers emphasize the need for further exploration. This research advances our understanding of BTM clinical applications in osteoporosis diagnosis and treatment.

## Introduction

The diagnostic criteria for osteoporosis, as defined by the World Health Organization(WHO), involve assessing bone mineral density (BMD) and determining if the measurement falls below 2.5 standard deviations (SD) from the reference mean of young women aged 20–29 years (T-score ≤ -2.5 SD) [1]. As per a report by the WHO, a substantial portion of patients experiencing osteoporotic fractures exhibits BMD measurements surpassing the diagnostic threshold (T value > -2.5) [2]. This indicates that BMD measurement represents the cumulative effect of prior bone loss before the assessment and exhibits limited sensitivity. Therefore, measuring multiple indicators is necessary to identify individuals who require prevention, treatment, or belong to the high-risk group for fractures, even without an osteoporosis diagnosis [3]. Theoretically, changes in BTMs should occur prior to bone loss and fractures. Therefore, BTMs can provide additional information, especially for patients with normal or decreased bone mass, where increased bone metabolism may be present without clinical symptoms. This suggests a potential increased risk of developing osteoporosis and osteoporotic fractures in the future [4]. The Bone Marker Standards Working Group (WG-BMS) of the International Osteoporosis Foundation (IOF) and the International Federation of Clinical Chemistry and Laboratory Medicine (IFCC) recommend P1NP(Procollagen type I N- propeptide ) and β-CTX (C-telopeptide cross-linked type 1 collagen) as reference markers, aiming to gather sufficient evidence in the future to incorporate BTMs alongside other risk factors in calculating fracture risk [5].

The utilization of peripheral blood measurements of BTMs to estimate bone turnover throughout the entire skeleton has its limitations. It’s important to recognize that BTMs present in peripheral blood do not exclusively originate from bone tissue. For example, while P1NP primarily derives from bone, a small amount also arises from other connective tissues like skin and tendons [6]. In adults, approximately 25% of trabecular bone undergoes resorption and remodeling annually, whereas cortical bone rebuilds at a much slower rate, around 3% [7]. Trabecular bone, which has a higher surface area-to-volume ratio compared to cortical bone, with 70-80% of its surfaces in contact with the bone marrow, plays a significant role in these processes. It’s worth noting that both osteoclasts and osteoblasts have their origins in the bone marrow, underscoring that a significant portion of bone remodeling occurs within the bone marrow environment [8].

Ornstrup et al. [9]conducted a 16-week study investigating the effects of resveratrol supplementation on BMD and BTMs. Their research unveiled that, at higher average concentrations, P1NP, β-CTX, and BAP exhibited substantial elevations in bone marrow compared to peripheral blood. In contrast, measurements of OC consistently demonstrated significantly lower levels in bone marrow compared to peripheral blood.

To explore potential discrepancies in BTMs levels between bone marrow and peripheral blood, examine the correlation between BTMs measurements in bone marrow and peripheral blood along with BMD, and determine significant differences in BTMs levels between patients with osteoporosis (T ≤ -2.5) and non-osteoporotic patients (T > -2.5), bone metabolism tests were conducted on bone marrow and peripheral blood samples obtained from 133 patients undergoing spinal surgery. The findings of our analysis confirmed notable disparities in BTMs levels between bone marrow and peripheral blood. Additionally, certain BTMs levels in both peripheral blood and bone marrow exhibited significant variations between patients with osteoporosis and non-osteoporotic patients.

## Materials and methods

### Study population

We conducted a retrospective study involving patients who underwent spinal surgery in our hospital between 2020 and 2022. The inclusion criteria were as follows: (1) patients who underwent spinal surgery(Percutaneous Vertebroplasty (PVP), Percutaneous Kyphoplasty (PKP), Endoscopic Discectomy, Laminectomy, Spinal Fusion.)due to severe thoracolumbar pain, lower limb paresthesia, distress, ambulatory challenges, and cauda equina syndrome, among other clinical manifestations and (2) patients with normal liver and kidney function test results. Conversely, the exclusion criteria encompassed: (1) patients with diseases causing secondary osteoporosis, such as primary hyperparathyroidism, hyperthyroidism, malabsorption disorders, celiac disease, systemic lupus erythematosus (SLE), (2) patients with other conditions that affect bone metabolism, including osteomalacia, Paget’s disease, metastatic bone tumors, chronic kidney disease, rheumatoid arthritis, multiple myeloma, hemophilia, inflammatory bowel disease (IBD), and (3) patients who received medications that impact bone metabolism, such as bisphosphonates, raloxifene, calcitonin, parathyroid hormone, antiepileptic drugs, aromatase inhibitors, and glucocorticoids. In total, 133 surgical patients, with an average age of 69.19 ± 8.36 years (all the women in the study were postmenopausal women), met the inclusion and exclusion criteria and were enrolled in this study. Based on lumbar spine BMD measurements, the patients were categorized into two groups: the osteoporosis group (T-score ≤ -2.5) and the non-osteoporosis group (T-score > -2.5). Ethical approval was obtained from the Medical Ethics Committee of Jiangsu Province Subei People’s Hospital (Ethics approval number:ky2020005), and informed consent was obtained from all participants prior to the commencement of the study, and the study was conducted in agreement with the Declaration of Helsinki II.

#### General patient information

Relevant demographic data, such as age, height, weight, and BMI, were gathered for all participants included in the study.

#### BMD

Preoperative BMD measurements were conducted on all participants using the GE “Prodigy Advance” dual-energy X-ray BMD machine. The scanner underwent daily calibration, ensuring the precision of DXA measurements at various anatomical sites, including L2-4, the total hip, and the femoral neck, with coefficient of variability (CV) values of 1.39%, 0.7%, and 2.22%, respectively. Additionally, the long-term reproducibility of DXA data throughout the trial, as determined through weekly repeated phantom measurements, exhibited a high level of consistency at 0.45%. Trained medical professionals from our hospital’s Medical Imaging Center performed the measurements. The BMD value of the lumbar spine (L2-L4) was utilized as the criterion for grouping.

#### BTMs

Fasting peripheral blood samples were collected from all participants within a strict time window between 6:00 and 7:00 in the morning, ensuring minimal biological variation due to the consistency in sampling time. These samples were promptly delivered to our hospital’s laboratory for the assessment of four peripheral blood BTMs: β-CTX, OC, P1NP, and 25(OH)D. Following collection, all blood samples were processed swiftly and stored at 3 °C to maintain their integrity until laboratory analysis. The process of collecting a bone marrow sample adhered to a rigorous surgical schedule. The patient was placed in a prone position, and the target area was meticulously disinfected with iodine, under aseptic conditions. Utilizing fluoroscopy from a C-arm machine, the exact location of the lesion in the vertebral body’s pedicle shadow was pinpointed. Subsequent incisions of approximately 0.5 cm were made on each side of the target area. A vertebral body shaping toolkit facilitated the insertion of a puncture needle up to the posterior edge of the vertebral body, followed by transitioning the sleeve to the middle of the vertebral body. After withdrawing the guide needle, bone marrow blood from the affected vertebral body was aspirated. The collected bone marrow specimens were immediately processed and preserved at 3 °C, similar to the blood samples, to ensure optimal sample integrity until their transport to the laboratory for further analysis. For control purposes, β-CTX, OC, P1NP, and 25(OH)D materials, sourced from Roche, a well-respected German manufacturer, were used. The analyses were conducted using the Cobas e602 automated chemiluminescence immunoassay analyzer, also provided by Roche. A precise centrifugation process was employed for obtaining the serum for bone turnover markers assessment, using a KDC-40 low-speed centrifuge from the China National Scientific Instruments and Materials Corporation. Specifically, a four-milliliter blood sample was centrifuged at 2191 × g for 10 min. The determination of β-CTX, OC, P1NP, and 25(OH)D exhibited intra-assay and inter-assay CVs of 2.5% and 3.5%, 2.9% and 4.0%, 2.3% and 2.8%, and 5.6% and 8.0%, respectively. All analyses of BTMs were conducted by proficient experts from the Department of Laboratory Medicine at the SuBei People’s Hospital in Jiangsu Province, China, ensuring the highest standards of accuracy and reliability.

### Statistical analysis

The statistical analysis was conducted using SPSS 23.0 software (SPSS, Inc., Chicago, Illinois, USA). All figures were created using Graphpad Prism9.To compare the variations in key characteristics between the non-osteoporotic and osteoporotic groups, we first assessed the distribution of age levels, which conformed to a normal distribution based on the Shapiro-Wilk test. However, it’s important to note that the distributions of height, weight, and BMI levels did not exhibit a normal distribution according to the Shapiro-Wilk test. As a result, we utilized an independent sample t-test to evaluate age differences between the two groups for normally distributed data, while for non-normally distributed data, we employed the Mann-Whitney U test. To compare the differences in BTMs between bone marrow and peripheral blood, the distribution of β-CTX levels followed a normal distribution (Shapiro-Wilk test, *P* = 0.053 > 0.05). However, the distributions of OC levels, P1NP levels, and 25(OH)D levels did not follow a normal distribution (Shapiro-Wilk tests, *P* = 0.000 < 0.05 for both). Thus, the paired sample t-test was employed to compare the differences in BTMs between the two groups for normally distributed data, while the Wilcoxon signed-rank test was used for non-normally distributed data. In the comparison of differences in BTMs between the non-osteoporosis group and the osteoporosis group, none of the BTMs followed a normal distribution (Shapiro-Wilk tests, *P* < 0.05 for all). Therefore, the Mann-Whitney U test was applied to compare the differences in BTMs between the two groups. The reported p-values in this study were two-tailed, and a significance level of *p* < 0.05 was considered statistically significant.

## Results

### Baseline characteristics

Table 1 displays the key characteristics of the entire participant cohort. Out of the 133 individuals included, all the women in the study were postmenopausal women.

**Table 1:** The key characteristics of the entire participant cohort

Variables	All subjects	non-osteoporosis group	osteoporosis group
Age(years)^a^	69.2 ± 8.4	68.3 ± 9.3	70.1 ± 7.2
Anthropometry			
Height(cm)^a^	160.0 ± 6.7	162.5 ± 7.0	157.2 ± 5.2
Weight (kg)^a^	60.6 ± 10.1	65.0 ± 8.3	56.0 ± 9.8
BMI (kg/m²)^a^	23.6 ± 3.4	24.7 ± 3.0	22.6 ± 3.5
gender			
men(%)^b^	16.5%	30.9%	1.5%
postmenopausal women(%)^b^	83.5%	69.1%	98.5%

Note: a Data expressed as mean ± standard deviation (SD), b Data expressed as a percentage

### Comparison of BTMs between bone marrow and peripheral blood

**Fig. 1:**
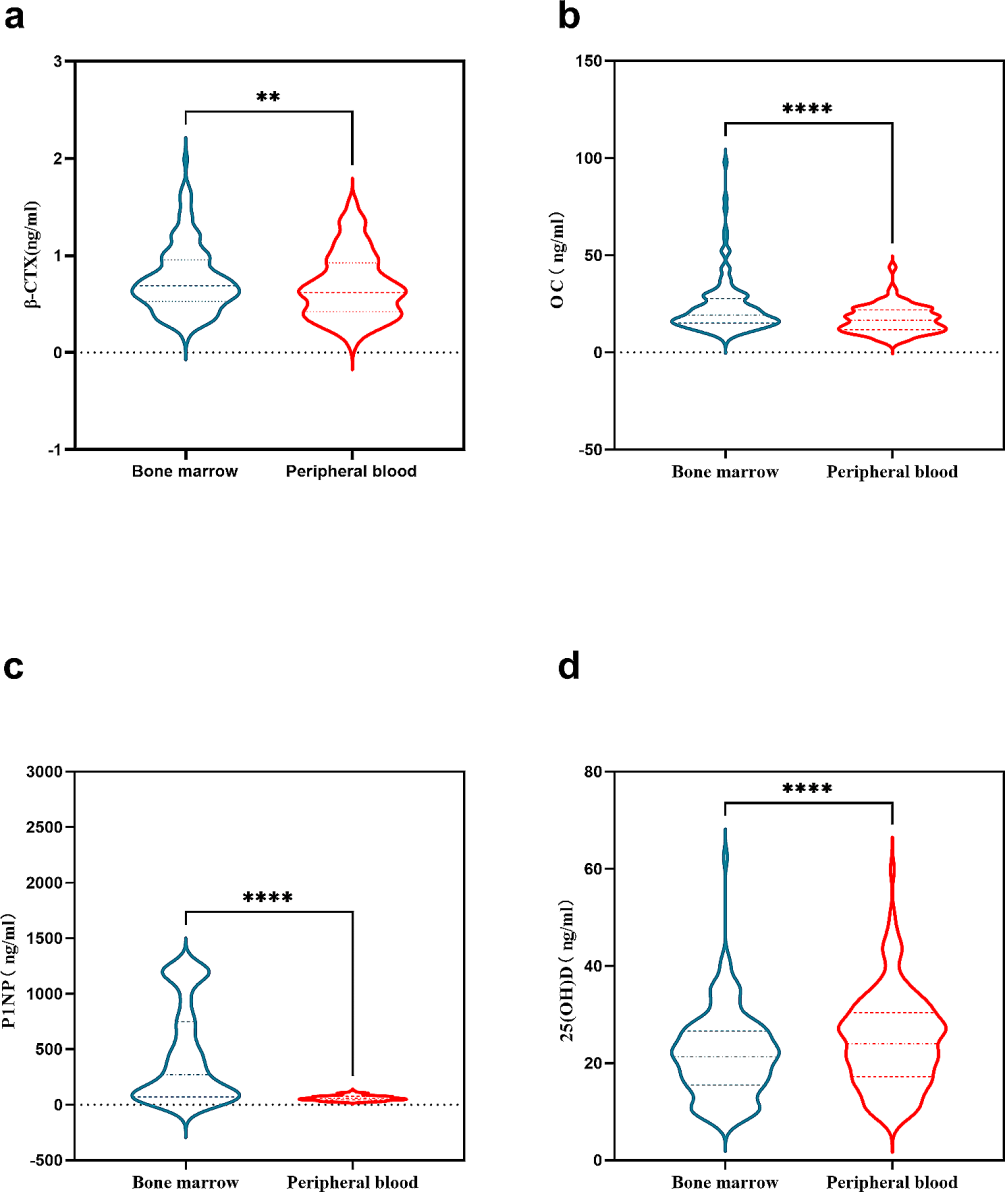
Paired sample t-test of β-CTX (panel **A**) and paired sample Wilcoxon signed-rank test of OC, P1NP and 25(OH)D (panel **B**, **C** and **D**). Note: ns: not significant, no significant difference; *: *p* < 0.05; **: *p* < 0.01; ***: *p* < 0.001; ****: *p* < 0.0001

**Fig. 2:**
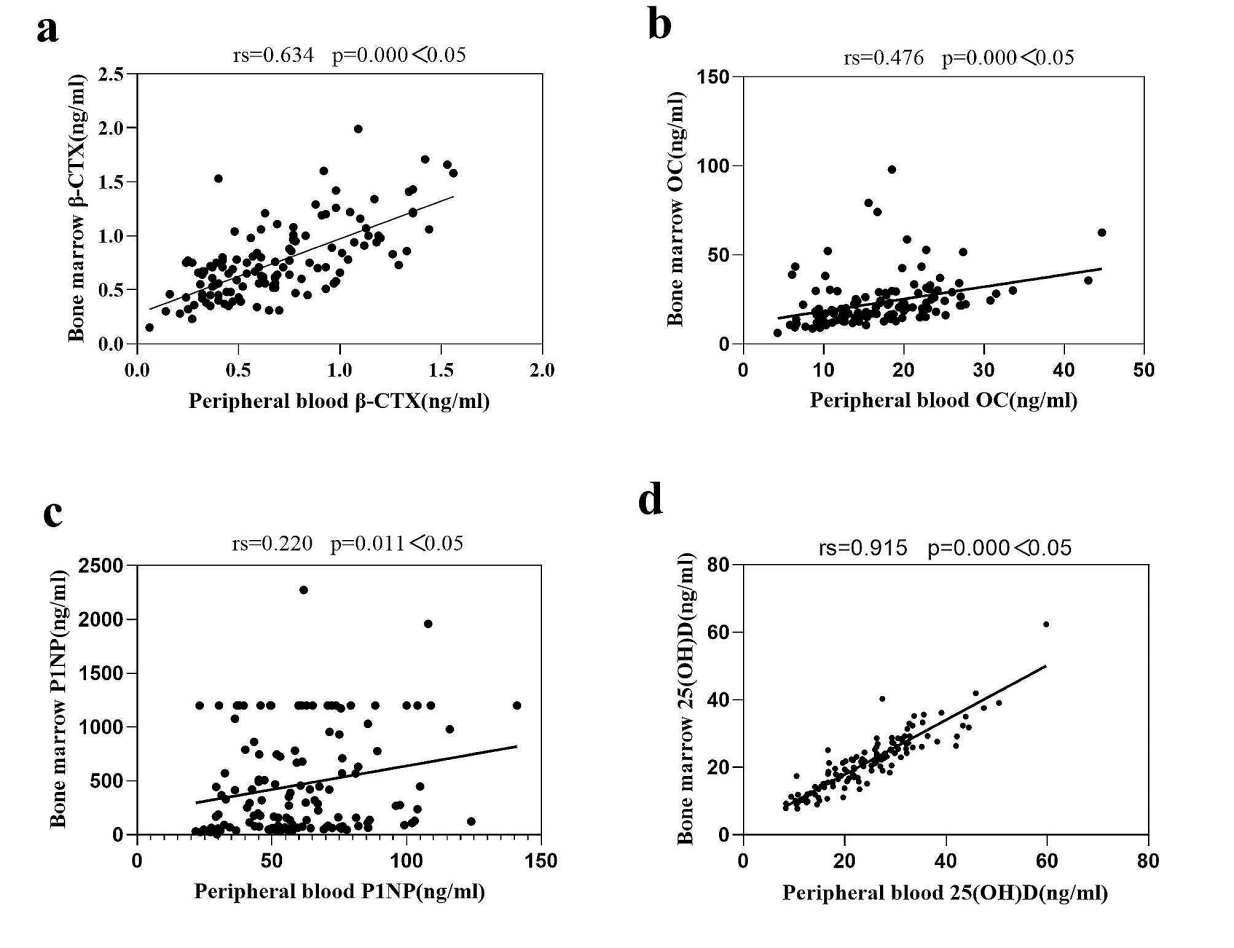
The bivariate Spearman correlation test between levels of bone markers in the peripheral blood and paired bone marrow

The paired sample t-test showed that the β-CTX levels in bone marrow was significantly higher than that in peripheral blood; the paired sample Wilcoxon signed-rank test revealed that the OC ,P1NP levels in bone marrow was significantly higher than that in peripheral blood and the 25(OH)D levels in bone marrow was significantly lower than that in peripheral blood (Table 2; Fig. 1). The levels of β-CTX, OC, P1NP and 25(OH)D in the peripheral blood have a positive correlation with paired bone marrow (Fig. 2).

**Table 2:** Comparing the levels of four BTMs between bone marrow and peripheral blood

	Bone marrow(*N* = 133)		Peripheral blood(*N* = 133)		
Variable	M	SD	M	SD	*p*
β-CTX (ng/ml)	0.765	0.341	0.708	0.321	0.005*
OC (ng/ml)	23.1	113.8	17.0	7.0	<0.0001**
P1NP (ng/ml)	467.1	473.7	60.5	23.4	<0.0001**
25(OH)D(ng/ml)	21.6	8.6	24.7	9.6	<0.0001**

Note: *: Paired sample t-test, **: Wilcoxon signed-rank test

In Fig. 1A, the three dashed lines within the graph represent the mean ± standard deviation of β-CTX levels. In Fig. 1B, C, and D, the three solid lines represent the quartiles of the corresponding datasets.

### Comparison of BTMs between the non-osteoporosis group and the osteoporosis group

**Fig. 3:**
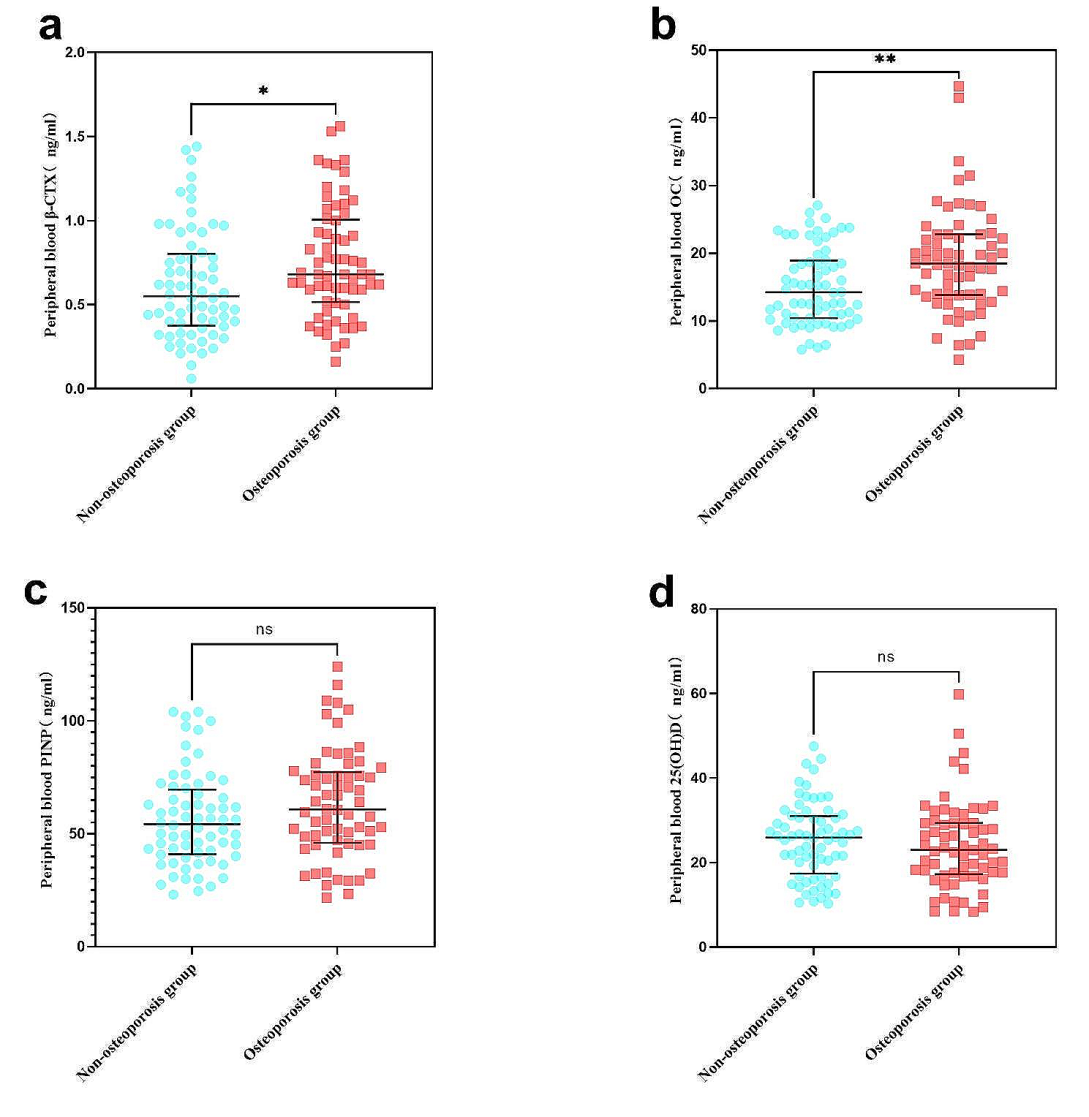
The non-parametric Mann-Whitney U test of peripheral blood CTX, OC, P1NP and 25(OH)D. Note: ns: not significant, no significant difference; *: *p* < 0.05; **: *p* < 0.01; ***: *p* < 0.001; ****: *p* < 0.0001. The three solid lines in each graph represent the quartiles of the data for each group

**Fig. 4:**
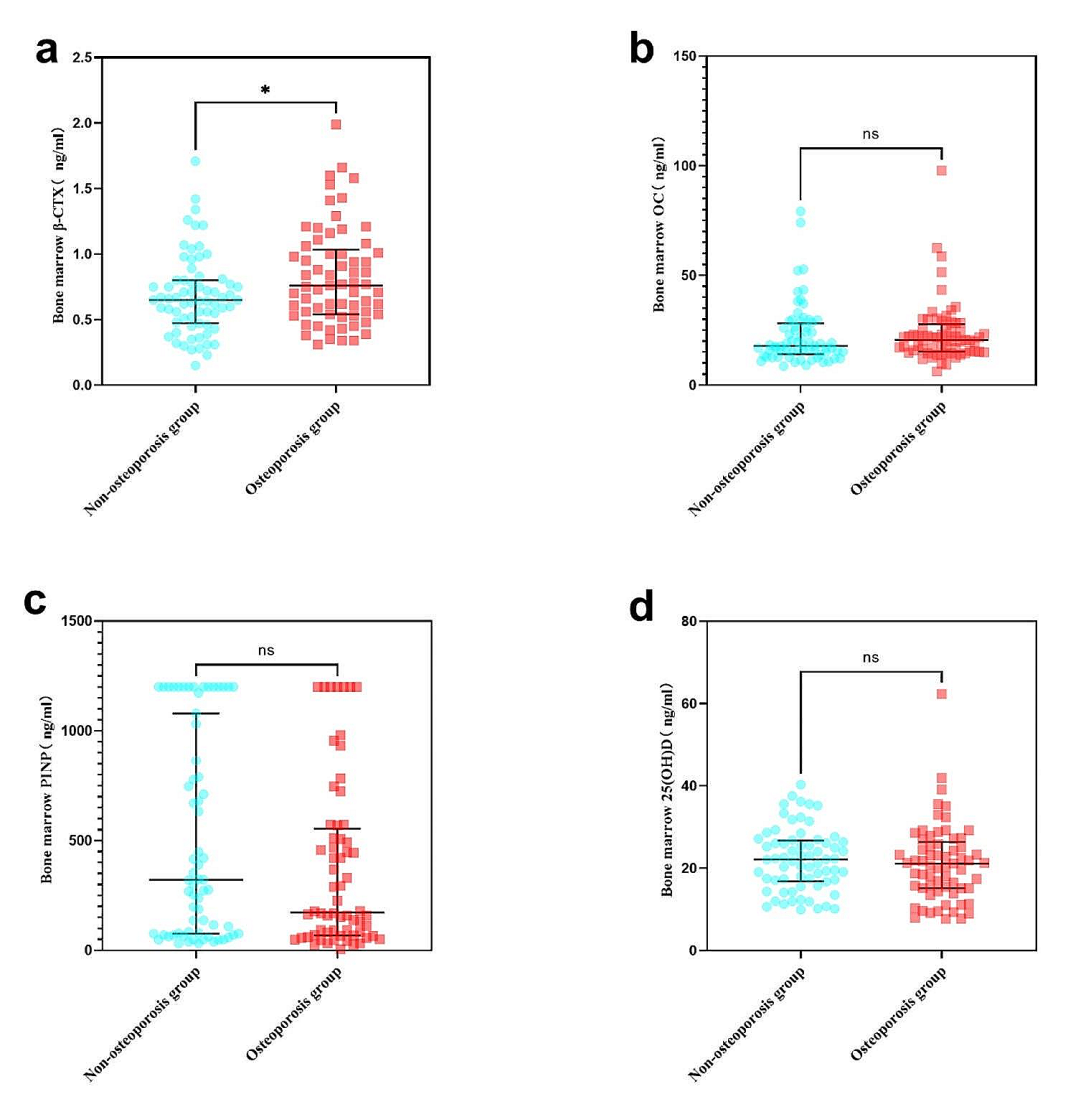
The non-parametric Mann-Whitney U test of bone marrow β-CTX, OC, P1NP and 25(OH)D. Note: ns: not significant, no significant difference; *: *p* < 0.05; **: *p* < 0.01; ***: *p* < 0.001; ****: *p* < 0.0001. The three solid lines in each graph represent the quartiles of the data for each group

The non-parametric Mann-Whitney U test revealed that the median values of peripheral blood β-CTX levels in the non-osteoporosis group were significantly lower compared to the osteoporosis group (Table 3; Fig. 3 Panel A). Similarly, the median values of bone marrow β-CTX levels in the non-osteoporosis group were significantly lower than in the osteoporosis group (Table 4; Fig. 4 Panel A).

Peripheral blood OC levels were lower in the non-osteoporosis group compared to the osteoporosis group (Table 3; Fig. 3 Panel B). However, there was no significant difference in bone marrow OC levels between the non-osteoporosis and osteoporosis groups (Table 4; Fig. 4 Panel B).

In Table 3; Fig. 3 Panel C and D, the non-parametric Mann-Whitney U test showed no significant differences between the non-osteoporosis group and the osteoporosis group in terms of peripheral blood P1NP levels and 25(OH)D levels. Similarly, there were no significant differences in bone marrow P1NP levels and 25(OH)D levels between the non-osteoporosis and osteoporosis groups (Table 4; Fig. 4 Panel C and D).

**Table 3:** Comparing the levels of Peripheral blood BTMs between the non-osteoporosis group and the osteoporosis group

	Non-osteoporosis group(*N* = 68)		Osteoporosis group(*N* = 65)		
Peripheral blood	M	SD	M	SD	*p*
β-CTX (ng/ml)	0.618	0.325	0.759	0.334	0.012
OC (ng/ml)	15.1	5.6	18.9	7.7	0.003
P1NP (ng/ml)	56.5	20.6	64.6	26.6	0.058
25(OH)D(ng/ml)	25.2	8.9	24.0	10.2	0.325

**Table 4:** Comparing the levels of bone marrow BTMs between the non-osteoporosis group and the osteoporosis group

	Non-osteoporosis group(*N* = 68)		Osteoporosis group(*N* = 65)		
Bone marrow	M	SD	M	SD	*p*
β-CTX (ng/ml)	0.685	0.303	0.830	0.372	0.034
OC (ng/ml)	22.7	13.7	23.7	14.1	0.302
P1NP (ng/ml)	531.8	508.0	399.8	428.2	0.21
25(OH)D(ng/ml)	22.1	7.6	21.2	9.5	0.347

### Correlations between various testing indicators and BMD

**Fig. 5:**
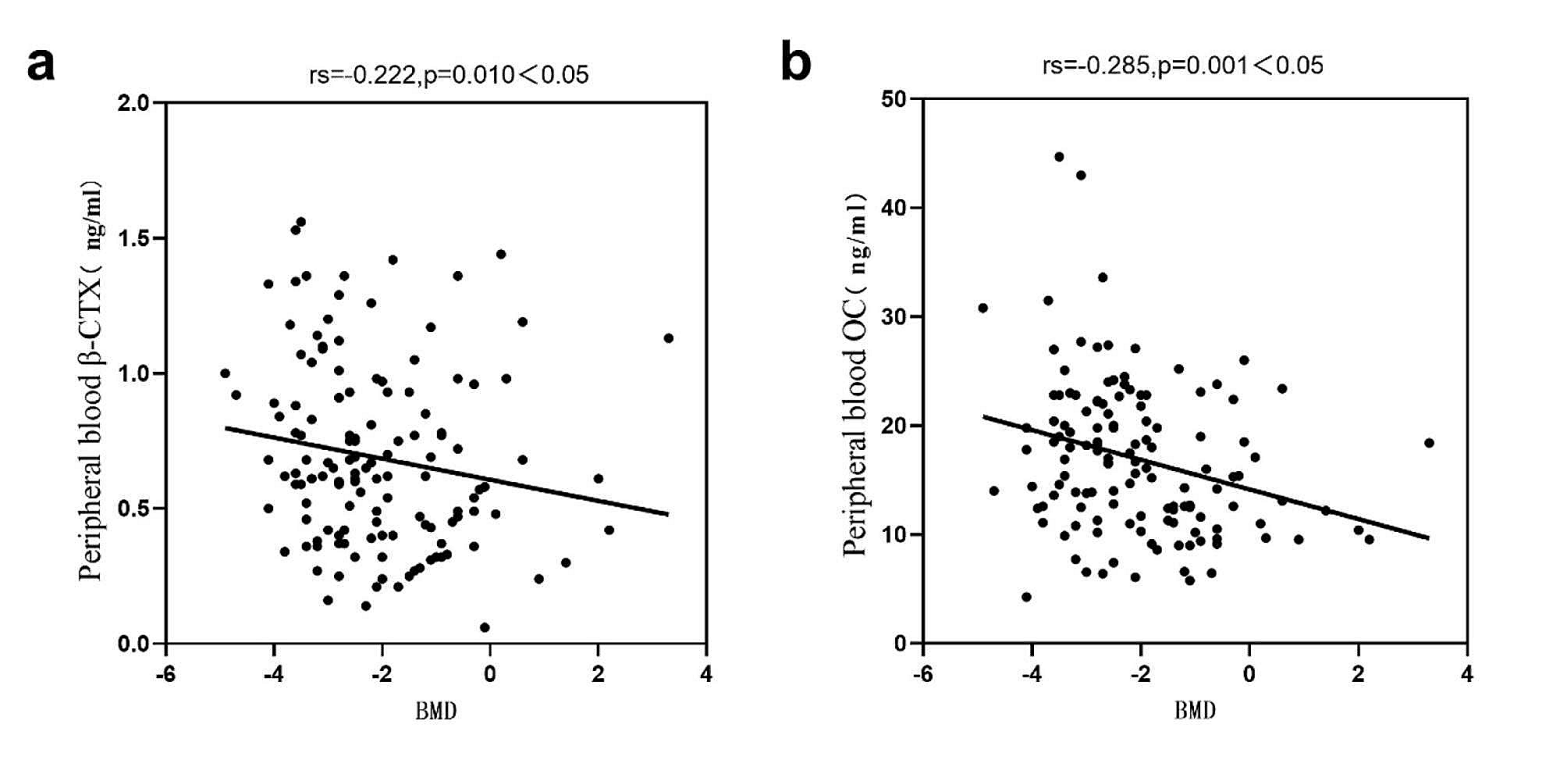
The bivariate Spearman correlation test between peripheral blood β-CTX, OC and BMD.

Table 5; Fig. 5 displays the correlations between different testing indicators and BMD. The bivariate Spearman correlation test reveals that peripheral blood β-CTX levels have a negative correlation with BMD, and peripheral blood OC levels also show a negative correlation with BMD. However, there are no significant correlations observed between the remaining testing indicators and BMD.

**Table 5:** The bivariate Spearman correlation test between different testing indicators and BMD.

Index	Rs	*P*
Peripheral blood β-CTX	-0.222*	0.010
Peripheral blood OC	-0.285**	0.001
Peripheral blood P1NP	-0.123	0.157
Peripheral blood 25(OH)D	0.053	0.547
Bone marrow β-CTX	-0.095	0.279
Bone marrow OC	-0.113	0.193
Bone marrow P1NP	0.133	0.126
Bone marrow 25(OH)D	0.060	0.493

Note: * Significantly correlated at the 0.05 level (two-tailed), ** Significantly correlated at the 0.01 level (two-tailed)

.

### Comparison of BTMs between bone marrow and peripheral blood in postmenopausal women

**Fig. 6:**
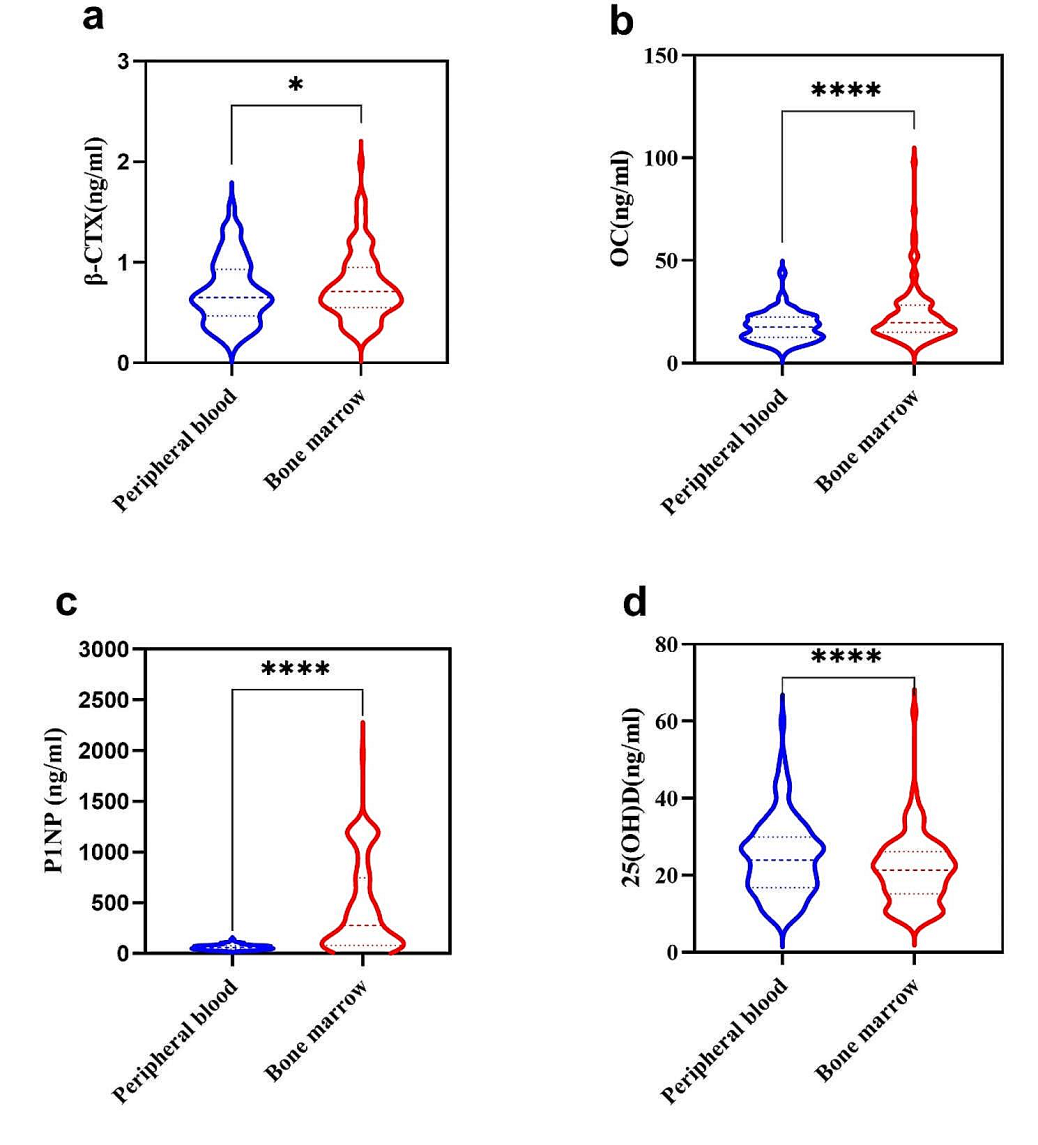
Paired sample t-test of β-CTX (panel **A**) and paired sample Wilcoxon signed-rank test of OC, P1NP and 25(OH)D (panel **B**, **C** and **D**) in postmenopausal women. Note: ns: not significant, no significant difference; *: *p* < 0.05; **: *p* < 0.01; ***: *p* < 0.001; ****: *p* < 0.0001

**Fig. 7:**
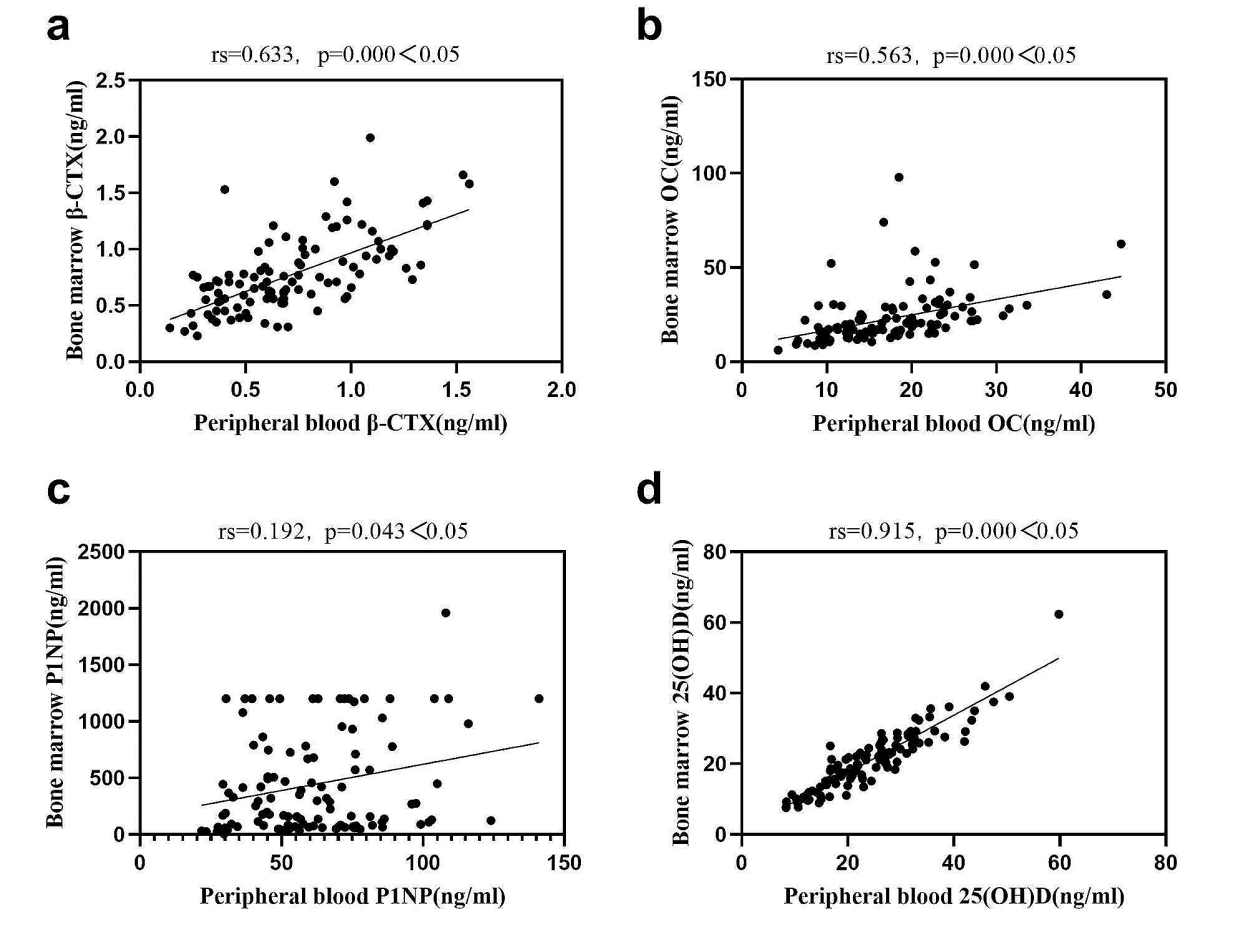
The bivariate Spearman correlation test between levels of bone markers in the peripheral blood and paired bone marrow in postmenopausal women

In postmenopausal women, the paired sample t-test showed that the β-CTX levels in bone marrow was significantly higher than that in peripheral blood; the paired sample Wilcoxon signed-rank test revealed that the OC, P1NP levels in bone marrow was significantly higher than that in peripheral blood and the 25(OH)D levels in bone marrow was significantly lower than that in peripheral blood (Table 6; Fig. 6). And the levels of β-CTX, OC, P1NP and 25(OH)D in the peripheral blood have a positive correlation with paired bone marrow (Fig. 7).

**Table 6:** Comparing the levels of four BTMs between bone marrow and peripheral blood in postmenopausal women

		Bone marrow(*N* = 133)			Peripheral blood(*N* = 133)		
Variable		M	SD		M	SD	*p*
β-CTX (ng/ml)		0.768	0.340		0.710	0.322	0.03*
OC (ng/ml)		23.0	13.6		17.7	7.0	<0.0001**
P1NP (ng/ml)		444.1	439.9		61.8	23.7	<0.0001**
25(OH)D(ng/ml)		21.2	8.7		24.6	9.8	<0.0001**

Note: *: Paired sample t-test, **: Wilcoxon signed-rank test

### Correlations between various testing indicators and BMD in postmenopausal women

**Fig. 8:**
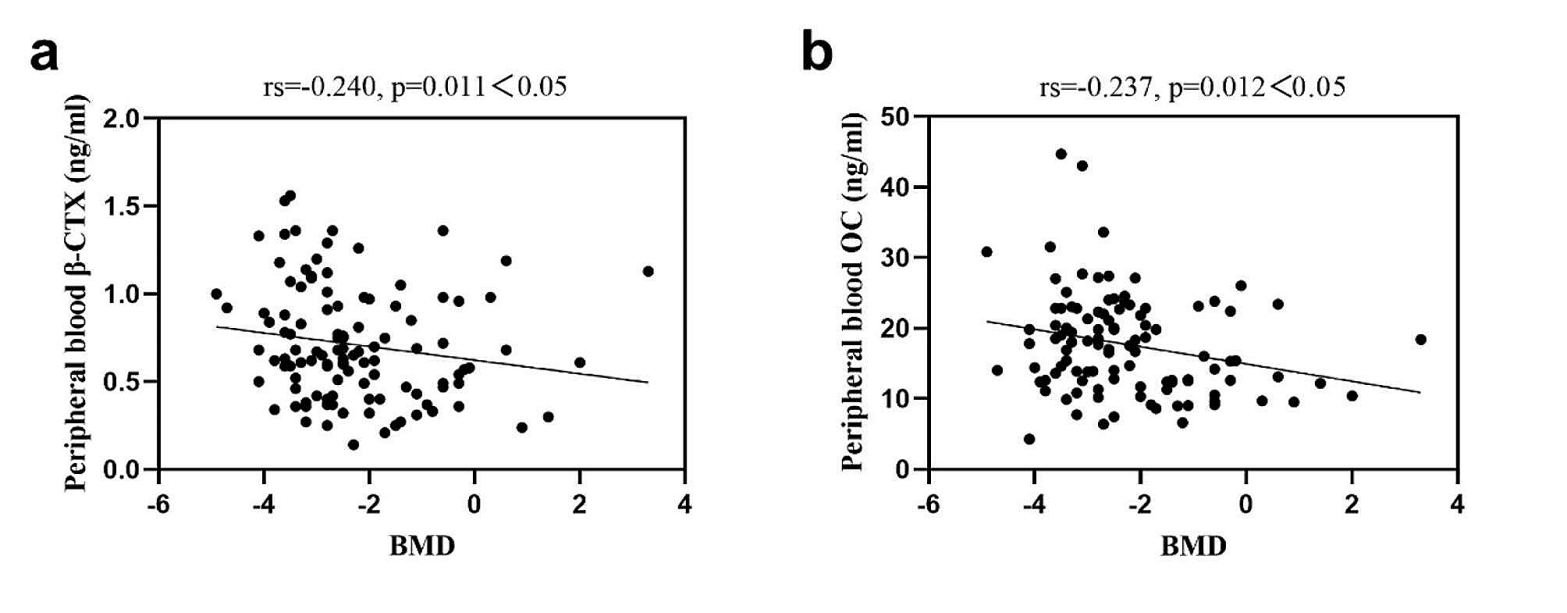
The bivariate Spearman correlation test between peripheral blood β-CTX, OC and BMD in postmenopausal women

Table 7; Fig. 8 displays the correlations between different testing indicators and BMD. The bivariate Spearman correlation test reveals that peripheral blood β-CTX levels have a negative correlation with BMD, and peripheral blood OC levels also show a negative correlation with BMD. However, there are no significant correlations observed between the remaining testing indicators and BMD.

**Table 7:** The bivariate Spearman correlation test between different testing indicators and BMD in postmenopausal women

Index	Rs	*P*
Peripheral blood β-CTX	-0.240*	0.011
Peripheral blood OC	-0.237*	0.012
Peripheral blood P1NP	-0.092	0.334
Peripheral blood 25(OH)D	0.017	0.862
Bone marrow β-CTX	-0.122	0.202
Bone marrow OC	-0.118	0.217
Bone marrow P1NP	0.155	0.105
Bone marrow 25(OH)D	-0.010	0.918

Note: * Significantly correlated at the 0.05 level (two-tailed)

## Discussion

In our research, we have observed that the levels of PINP, β-CTX, OC, and 25(OH)D in bone marrow were significantly higher than those in peripheral blood, an observation that aligns with the theoretical suggestion by Ornstrup [9]. and others that bone remodeling should stimulate the production and release of these BTMs, leading to higher levels in bone marrow. However, a notable discrepancy emerged when comparing our results with Ornstrup’s study, particularly regarding OC levels. While we found substantially higher OC levels in bone marrow, Ornstrup reported lower levels in bone marrow compared to peripheral blood. This intriguing disparity merits further exploration into the factors influencing BTM levels. One significant factor contributing to this discrepancy could be the variations in bone marrow collection sites. Ornstrup’s research focused on bone marrow from the posterior iliac crest, whereas our study utilized samples from the thoracolumbar region. This distinction is critical as weight-bearing regions like the thoracolumbar spine might exhibit higher BTM turnover and release, potentially explaining the elevated BTM levels in our findings. Additionally, our results contribute a new perspective to the current understanding of BTMs in osteoporosis. The marked difference in OC levels between bone marrow and peripheral blood in our study underscores the complexity of bone metabolism and its clinical implications. It highlights the need for a comprehensive approach in studying bone metabolism, taking into account factors such as sampling site and patient demographics. Moreover, these findings emphasize the importance of expanding research methodologies in future studies. Investigating BTMs across various bone marrow sites and in diverse patient populations could provide a more holistic understanding of bone metabolism in osteoporosis. This approach is invaluable for refining diagnostic criteria and developing targeted therapies for osteoporosis and related bone diseases. Furthermore, existing research, including studies exploring the role of metabolic factors such as metabolic syndrome, provides valuable context to our findings. For instance, studies like those conducted by Ornstrup have delved into how metabolic conditions might influence BTM levels in bone marrow. This body of research offers insights that complement our own observations, suggesting a complex interaction between metabolic health and bone turnover processes. Our study contributes to this broader discussion by focusing on different BTMs in bone marrow and peripheral blood, thereby enhancing our understanding of bone metabolism in varying physiological conditions.

In conclusion, our research not only contributes to the ongoing discourse on the optimal use of BTMs in osteoporosis research but also underscores the necessity for standardized protocols in sample collection. The importance of considering anatomical and patient-specific factors in research design is paramount, ultimately contributing to a nuanced understanding of bone health and disease. We also observed significant differences in peripheral blood β-CTX and OC levels between the osteoporosis group and the non-osteoporosis group, with higher levels detected in the osteoporosis group, suggesting their potential as indicators for the presence of osteoporosis. However, only β-CTX levels showed significant differences in the bone marrow between the two groups, while OC levels did not exhibit significant differences. OC is a bone-specific protein consisting of 49 amino acids and represents the most abundant non-collagenous bone protein [9, 10]. Newly formed OC in osteoblasts is released, with some being incorporated into bone and some entering circulation [11]. One possible explanation for our findings is that the relatively small size of the OC protein molecule allows for easier entry into peripheral circulation from the bone marrow, resulting in no significant difference in bone marrow OC levels between the osteoporosis and non-osteoporosis groups. However, this is mere speculation and lacks sufficient evidence for a conclusive causal explanation. Further research is required to explore the underlying mechanisms.

To understand the relationship between BTM levels in the bone marrow and peripheral blood with BMD, we investigated their associations at the L2 to L4 vertebrae. BTMs measured in the bone marrow showed no association with BMD, while peripheral blood β-CTX and OC levels demonstrated a negative correlation with BMD. These findings suggest that bone marrow BTMs may be less effective predictors of BMD compared to BTMs derived from peripheral blood. During the correlation analysis of BMD with bone marrow samples (β-CTX, OC, P1NP, 25(OH)D), it’s essential to consider the potential influence of variability within the groups on the strength and significance of these correlations. Subgroups within the β-CTX, OC, P1NP, or 25(OH)D categories might exhibit distinct relationships with BMD. To address this, we conducted a separate analysis specifically focused on the correlation between bone marrow samples from postmenopausal women and BMD, and the results align with those obtained from the overall study population. Moreover, our study participants originate from the same region and share minimal differences in terms of age, developmental stages, environment, and lifestyle factors. We meticulously excluded individuals with relevant diseases and medication effects during the enrollment process. Nevertheless, it is imperative to recognize the role of uncontrollable genetic and biological variations in contributing to disparities in bone turnover rates. Moreover, the complex interplay among BTMs could significantly influence their efficacy in BMD analyses and their associative relationships. In addressing the challenges unearthed in our current research, particularly the saturation phenomenon observed at P1NP levels around 1200 ng/ml, our forthcoming studies will be directed towards several critical improvements. Initially, we plan to integrate measurement methodologies that encompass a more extensive detection range. This strategy is specifically devised to overcome the limitations associated with P1NP level saturation, thereby facilitating a more precise assessment and enabling comparisons across a wider spectrum of concentrations. In addition, a more profound examination of our samples is anticipated to identify specific physiological or pathological conditions that may precipitate abnormally high P1NP levels.

Biological variations significantly influence the levels of BTMs and can potentially impact their clinical interpretation. These variations encompass factors such as circadian rhythm, growth, aging, dietary calcium intake, gender, and hormonal status [12]. BTM levels measured in urine are particularly affected by substantial biological variation, limiting their clinical applicability [13]. Conversely, BTMs measured in blood exhibit significantly lower biological variation [14]. In our study, serum samples were utilized for BTM measurement. It is worth noting that certain BTMs, like β-CTX, are influenced by meal conditions and circadian rhythm, necessitating blood sample collection following an overnight fast [15]. On the other hand, the levels of PINP in blood display slight circadian rhythm variation, and the impact of food intake on PINP levels is minimal, enabling testing at any time during the day [16]. OC, similar to other BTMs, exhibits circadian rhythm variation but remains unaffected by food intake [17]. In our study, the assessment of peripheral blood BTMs strictly adhered to the protocol of an overnight fast and blood sample collection between 6:00 and 7:00 in the morning. However, the evaluation of bone marrow BTMs required blood sample collection based on participants’ surgical times. As a result, some participants had morning blood samples, while others had samples collected in the afternoon or evening. The lack of strict consistency in the collection time for bone marrow samples is a limitation of this study.

The increasing clinical application of BTMs in conditions such as osteoporosis and malignant tumor bone metastasis underscores the necessity to address clinical gaps through substantial long-term research. While peripheral blood BTMs have shown potential in predicting fracture risk, assessing patient response to osteoporosis treatment, and monitoring the progression of bone metastasis, the complete spectrum of their utility is yet to be fully established. This calls for rigorous research to define reference intervals, treatment targets, and the overall effectiveness of BTMs in these clinical settings. In our study, significant differences were observed in specific BTMs, with notably higher levels in bone marrow compared to peripheral blood. This finding highlights the potential of bone marrow as a critical site for the study of bone metabolism, particularly in the context of osteoporosis. The choice of bone marrow samples, despite its invasive nature, was driven by the need to explore deeper into their role as biomarkers. However, we acknowledge that the selectivity of this approach, necessitated by the invasive sample collection method, may limit the general applicability of our findings. As such, our study primarily focuses on a specific patient group, especially those who have undergone bone marrow extraction for other medical reasons. Looking ahead, it is imperative to explore non-invasive methods for assessing BTMs in bone marrow. This could involve the development of new biomarkers or the refinement of existing detection techniques. As research in this field advances, we anticipate broader application of our findings, significantly benefiting clinical practice, particularly in the diagnosis and treatment of osteoporosis. Despite the current limitations in collecting bone marrow samples, ongoing research is expected to reveal bone marrow BTMs with less biological variation and higher sensitivity. Such advancements will provide valuable data for clinical practice and contribute to the development of more effective treatment strategies for osteoporosis and related conditions.

Subsequent investigations could be strategically designed to elucidate the variations in BTM profiles in the context of diverse bone pathologies, including osteoarthritis and bone malignancies. Furthermore, a longitudinal analysis of these markers, tracking their temporal evolution post-bone injury or throughout the progression of bone pathologies, holds significant promise in unravelling the intricate biological processes that govern bone remodeling and repair. Expanding the ambit of our research to encompass these dimensions will substantially deepen our comprehension of BTMs. This expanded understanding is poised to augment their applicability in clinical diagnostics and therapeutics, ultimately culminating in enhanced patient outcomes for osteoporosis and a spectrum of other bone-related disorders.

## Data Availability

No datasets were generated or analysed during the current study.
